# Dataset on growth curves of Boer goats fitted by ten non-linear functions

**DOI:** 10.1016/j.dib.2019.01.020

**Published:** 2019-01-15

**Authors:** J.G. García-Muñiz, R. Ramírez-Valverde, R. Núñez-Domínguez, J.A. Hidalgo-Moreno

**Affiliations:** Universidad Autónoma Chapingo, Departamento de Zootecnia, Posgrado en Producción Animal, km 38.5 Carretera México-Texcoco, Chapingo, Estado de México 56230, México

**Keywords:** Growth curve models, Boer goats, Goodness of fit

## Abstract

Data on the description of growth of female Boer goats from the Mexican national breeding flock are presented. Goat meat is highly appreciated for the preparation of traditional dishes of Mexican cuisine, and its demand is on the rise. Boer goats are of relatively recent arrival in Mexico and the size of the performance-recorded flock has been increasing steadily in the last ten years. Repeated measures of body weight at different ages from birth to adulthood of Boer goats are scarce. When available, such data can be used to describe the growth pattern and the meat production potential of goat meat breeds such as the Boer. This paper presents data on estimators of growth curve parameters, plots of average predicted growth curves, plots of residuals on age, and data on goodness of fit statistics of ten non-linear functions fitted to describe the growth curve of Boer goats.

**Specifications table**

**Table:** 

Subject area	*Agricultural and Biological Sciences*
More specific subject area	*Animal sciences, growth curve modeling of small ruminants*
Type of data	*Tables and figures*
How data was acquired	*For the 2017 genetic evaluation of the Boer breed in Mexico, animal unique identification, sex, birth date, date of weight recording, body weight records, and age at weight recording, were obtained from the national database during the data edition phase.*
Data format	*Processed, analyzed*
Experimental factors	*Only data from females with valid individual identification number, known farm of origin, known birth date, and with three or more weight records were included in the analysis.*
Experimental features	*Ten non-linear functions were fitted to the same dataset comprising age-weight records of individual Boer goats from the National Breeding Flock to produce growth curve parameters, goodness of fit estimators, plots of predicted average growth curves and plots of residuals.*
Data source location	*Laboratorio de Evaluaciones Genéticas, Universidad Autónoma Chapingo, Departamento de Zootecnia, Posgrado en Producción Animal, km 38.5 carretera México-Texcoco, Chapingo, Estado de México.*
Data accessibility	*Data is with this article.*
Related research article	*García-Muñiz J.G., Ramírez-Valverde R., Núñez-Domínguez R., Hidalgo-Moreno J.A. 2018.* Genetic parameters for direct and maternal effects on accumulated productivity to weaning of Boer goats in Mexico. *Proceedings of the World Congress on Genetics Applied to Livestock Production, 11.92*

**Value of the data**

• Small ruminants such as meat goats are expected to play an increasingly important role in food production worldwide.
• Efficient meat production requires appropriate description of the growth pattern of populations of animals.
• Data required to describe the growth trajectory of female goats may be available from national databases used for genetic evaluation. Often, however, this information needs to be compiled and edited in an appropriate format to undertake growth curve analysis.
• National datasets with repeated weight records of female Boer goats covering the interval from birth to maturity are scarce. Thus, the data presented may be used with sets of similar data to compare the growth pattern of Boer goats from different populations.

## 1 Data

**Fig. 1:**
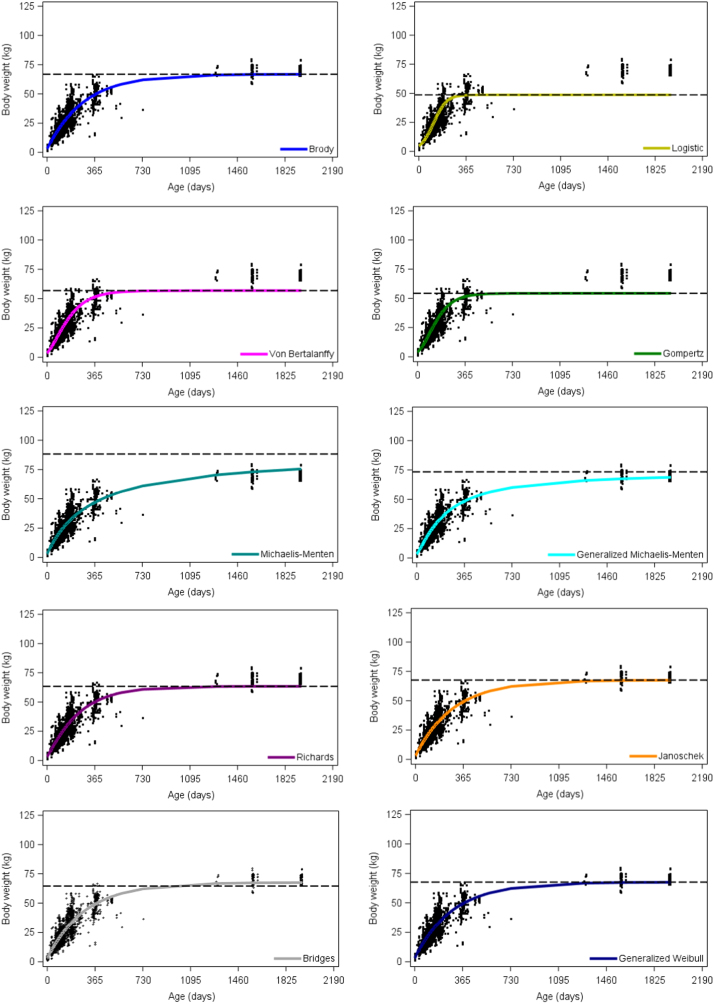
Visual display of average fixed growth curves of Boer goats fitted by 10 non-linear functions. The horizontal broken line corresponds to the estimated asymptotic (mature) body weight, and the black dots are observed weights.

Weight-age records (*n* = 3783) and animal unique identification number, birth date, date of weight recording, farm of origin and farm location were obtained from the national database of the Boer goat breed in Mexico (Appendix A). Data represented females (*n* = 1055) born from 2006 to 2016, that belonged to 31 farms distributed across 8 states in the country. The animal and farm variables recorded represented the variability of the national Boer flock (Table 1). For the growth interval from 1 to 1944 days of age, scatter plots of sigmoid trajectories of weight-age data (Fig. 1) along with the estimated average growth curve, and the asymptotic (mature) weight are displayed for each of 10 non-linear functions fitted to the data.

**Table 1:** Descriptive statistics for age, weight and number of records per farm and per animal of female Boer goats from the Mexican national breeding flock.

Variable	*N*	Minimum	Maximum	Mean	Std. Dev.
Age (days)	3783	1.0	1944.0	159.0	315.1
Weight (kg)	3783	1.2	80.2	21.5	17.7
Weight-age records per animal (n)	1055	3.0	5.0	3.6	0.7
Weight-age records per farm (n)	3783	12.0	865.0	122.0	172.7

## 2 Experimental design, materials and methods

### 2.1 Description of models fitted

Ten non-linear functions describing sigmoid trajectories of age-weight data were fitted separately to the same data set (Table 2).

**Table 2:** Description of models and model re-parameterizations fitted to describe the growth curve of Boer goats from the Mexican national breeding flock.

Model	Reference	Model general form	Expression for ***b***	Model׳s re-parameterization fitted
Brody	Fitzhugh [1]	$W_{t}=A{\left(1-be^{-\mathrm{kt}}\right)}^{1}$	$1-\frac{W_{0}}{A}$	$W_{t}=A\left[1-\left(1-\frac{W_{0}}{A}\right)e^{-\mathrm{kt}}\right]$
Logistic	Tjørve and Tjørve [2]	$W_{t}=A{\left(1+be^{-\mathrm{kt}}\right)}^{-1}$	$\frac{A}{W_{0}}-1$	$W_{t}=A{\left[1+\left(\frac{A}{W_{0}}-1\right)e^{-\mathrm{kt}}\right]}^{-1}$
Von Bertalanffy	Tjørve and Tjørve [2]	$W_{t}=A{\left(1-be^{-\mathrm{kt}}\right)}^{3}$	$1-{\left(\frac{W_{0}}{A}\right)}^{1/3}$	$W_{t}=A{\left\{1-\left[1-{\left(\frac{W_{0}}{A}\right)}^{\frac{1}{3}}\right]e^{-\mathrm{kt}}\right\}}^{3}$
Gompertz	Tjørve and Tjørve [3]	$W_{t}=Ae^{-be^{-\mathrm{kt}}}$	$\ln\left(\frac{A}{W_{0}}\right)$	$W_{t}=A{\left(\frac{W_{0}}{A}\right)}^{e^{-\mathrm{kt}}}$
Michaelis-Menten	López et al. [4]	$W_{t}=\frac{W_{0}K+\mathrm{At}}{K+t}$	–	$W_{t}=\frac{W_{0}K+\mathrm{At}}{K+t}$
Generalized Michaelis-Menten	López et al. [4]	$W_{t}=\frac{W_{0}K^{n}+At^{n}}{K^{n}+t^{n}}$	–	$W_{t}=\frac{W_{0}K^{n}+At^{n}}{K^{n}+t^{n}}$
Richards	Tjørve and Tjørve [2]	$W_{t}=A{\left(1-be^{-\mathrm{kt}}\right)}^{n}$	$1-{\left(\frac{W_{0}}{A}\right)}^{1/n}$	$W_{t}=A{\left\{1-\left[1-{\left(\frac{W_{0}}{A}\right)}^{1/n}\right]e^{-\mathrm{kt}}\right\}}^{n}$
Janoschek	Wellock et al. [5]	$W_{t}=A-\left(A-b\right)e^{-kt^{n}}$	$W_{0}$	$W_{t}=A-(A-W_{0})e^{-kt^{n}}$
Bridges	Wellock et al. [5]	$W_{t}=W_{0}+A(1-e^{-kt^{n}})$	–	$W_{t}=W_{0}+A(1-e^{-kt^{n}})$
Generalized Weibull	Henderson and Seaby [6]	$W_{t}=A\left[1-be^{-{\left(\mathrm{kt}\right)}^{n}}\right]$	$1-\frac{W_{0}}{A}$	$W_{t}=A\left[1-\left(1-\frac{W_{0}}{A}\right)e^{-{\left(\mathrm{kt}\right)}^{n}}\right]$

The first five models from Table 2 are models with three-parameters (***A***, ***W***_***0***_, and ***k***), whereas the last five, are models that include a fourth parameter (***A***, ***W***_***0***_, ***k***, and ***n***). Except for the models of Bridges, and the Generalized Michaelis-Menten, that both have initial weight ($W_{0}$) as a parameter, the remaining models were reparameterized to contain $W_{0}$ as a model parameter. To achieve this end, the procedure of Koya and Goshu [7] was followed to obtain an expression for the *b* parameter of the model general form. The re-parameterized version of the model was obtained by substituting *b* by its expression in the model general form (Table 2). Each of the ten models containing $W_{0}$ as a parameter was fitted separately to the dataset using the NLMIXED procedure of SAS [8]. For the ten models fitted, only the parameter related to asymptotic weight (***A***) was fitted as a random effect. Thus, the statistical model fitted for each of the growth functions compared can be expressed as follows:

(1) $$\mathrm{Brody}\;:\;W_{i,t}=(A+a_{i})\left(1-be^{-\mathrm{kt}}\right)+\varepsilon_{i,t}$$

(2) $$\mathrm{Logistic}\;:\;W_{i,t}=\left(A+a_{i}\right){\left[1+\left(\frac{A+a_{i}}{W_{0}}-1\right)e^{-\mathrm{kt}}\right]}^{-1}+\varepsilon_{i,t}$$

(3) $$\mathrm{Von\backslash Bertalanffy}\;:\;W_{i,t}=\left(A+a_{i}\right){\left\{1-\left[1-{\left(\frac{W_{0}}{A+a_{i}}\right)}^{\frac{1}{3}}\right]e^{-\mathrm{kt}}\right\}}^{3}+\varepsilon_{i,t}$$

(4) $$\mathrm{Gompertz}\;:\;W_{i,t}=\left(A+a_{i}\right){\left(\frac{W_{0}}{A+a_{i}}\right)}^{e^{-\mathrm{kt}}}+\varepsilon_{i,t}$$

(5) $$\mathrm{Michaelis}-\mathrm{Menten}\;:\;W_{i,t}=\frac{W_{0}K+\left(A+a_{i}\right)t}{K+t}+\varepsilon_{i,t}$$

(6) $$\mathrm{Generalized\backslash Michaelis}-\mathrm{Menten}\;:\;W_{i,t}=\frac{W_{0}K^{n}+\left(A+a_{i}\right)t^{n}}{K^{n}+t^{n}}+\varepsilon_{i,t}$$

(7) $$\mathrm{Richards}\;:\;W_{i,t}=\left(A+a_{i}\right){\left\{1-\left[1-{\left(\frac{W_{0}}{A+a_{i}}\right)}^{1/n}\right]e^{-\mathrm{kt}}\right\}}^{n}+\varepsilon_{i,t}$$

(8) $$\mathrm{Janoscheck}\;:\;W_{i,t}=A+a_{i}-\left[\left(A+a_{i}\right)-W_{0}\right]e^{-kt^{n}}+\varepsilon_{i,t}$$

(9) $$\mathrm{Bridges}\;:\;W_{i,t}=W_{0}+\left(A+a_{i}\right)(1-e^{-kt^{n}})+\varepsilon_{i,t}$$

(10) $$\mathrm{Generalized\backslash Weibull}\;:\;W_{i,t}=\left(A+a_{i}\right)\left[1-\left(1-\frac{W_{0}}{A+a_{i}}\right)e^{-{\left(\mathrm{kt}\right)}^{n}}\right]+\varepsilon_{i,t}$$

Where *W*_*i,t*_ is body weight of animal *i* recorded on day *t* of age, *e* is the base of natural logarithms (i.e. 2.718281), ***A*** is the predicted mature (asymptotic) weight, *a*_*i*_ is the random effect of animal *i* for the parameter ***A*** of the growth curve $\sim \mathrm{Normal}\left(0,\sigma_{A}^{2}\right)$, *t* is age in days, $W_{0}$ is initial (birth) weight (kg), *k* is maturation rate, *n* is an inflection parameter, and ε_i,t_ is the residual $\sim \mathrm{Normal}\left(0,\sigma_{\varepsilon }^{2}\right)$. The terms *W*_*i,t*_ and the parameters ***A*** and $W_{0}$ of the Michaelis-Menten and its generalized equation are as described before. For these two functions the parameter *K* represents the time (days) at which 50% of total asymptotic weight is achieved [4]. Models were fitted iteratively and initial values were given for the parameters of the growth curve, ***A*** (from 50 to 100 by 10), $W_{0}$ (from 1 to 5 by 0.5), *k* (from 0.0001 to 0.0005 by 0.0001), *n* (from 1 to 5 by 1), $\sigma_{A}^{2}=550$, $\sigma_{\varepsilon }^{2}=15$. Bounds were established for $\sigma_{A}^{2}$ > 0 and for $\sigma_{\varepsilon }^{2}$ > 0. The double-dogleg optimization method was specified (method=DBLDOG) and the number of iterations was set to 200.

### 2.2 Goodness of fit estimators

At model convergence, the fitting of these functions generated estimators for the parameters describing the growth curve (Table 3), estimators for age and weight at inflection (Table 4) using notation derived by Goshu and Koya [9], estimators of variance ($\sigma_{A}^{2}$) for the ***A*** parameter and the residual variance (${\text{σ}}_{\varepsilon }^{2}$) (Table 5), and the goodness of fit estimators -2 Log Likelihood, Akaike Information Criterion (AIC) and Bayesian Information Criterion (BIC) generated by the NLMIXED procedure of SAS [8]. The standard error of the regression (Sy/x) was calculated as an additional goodness of fit criteria for each of the models fitted (Table 6), using the following expression:

$$S_{y/x}=\sqrt{\frac{1}{n-p}\sum_{t=1}^{n}e_{t}^{2}}$$

Where, in this case, *n* is the number of age-weight observations in the data set; *p* is the number of parameters estimated by the model; $e_{t}^{2}$ are the squared deviations of the observed minus the average predicted weight of the respective model fitted.

**Table 3:** Growth curve parameters estimated for ten non-linear functions fitted to describe the growth curve of Boer goats from the Mexican national breeding flock.

Model	Parameter^a^	Estimate	Std. Error	95% Confidence Limits	
Brody	*A*	67.3	0.6558	66.0	68.6
	$W_{0}$	3.05	0.0862	2.88	3.22
	*k*	0.00354	4.8E−5	3.4E−3	3.6E−3
Logistic	*A*	49.5	0.5147	48.5	50.5
	$W_{0}$	4.84	0.0631	4.71	4.96
	*k*	0.01931	1.6E−4	0.0190	0.0196
Von Bertalanffy	*A*	57.4	0.5121	56.4	58.4
	$W_{0}$	3.69	0.0731	3.55	3.83
	*k*	0.00783	7.5E−5	0.0077	0.0080
Gompertz	*A*	54.7	0.5027	53.7	55.7
	$W_{0}$	3.98	0.0679	3.85	4.12
	*k*	0.01047	9.1E−5	0.0103	0.0106
Michaelis-Menten	*A*	88.5	1.0122	86.5	90.5
	$W_{0}$	2.84	0.0862	2.67	3.00
	*K*	338.1	5.8621	326.6	349.6
Generalized Michaelis-Menten	*A*	71.8	1.0576	69.7	73.9
	$W_{0}$	3.45	0.0913	3.27	3.63
	*K*	213.5	5.6034	202.5	224.5
	*n*	1.2813	0.0221	1.2379	1.3248
Richards	*A*	59.5	0.6828	58.1	60.8
	$W_{0}$	16.35	1.3197	13.8	18.9
	*k*	0.00609	1.8E−4	5.7E−3	6.4E−3
	*n*	1.6603	0.0568	1.5489	1.7718
Janoscheck	*A*	67.1	0.9149	65.3	68.9
	$W_{0}$	3.06	0.0967	2.8704	3.2497
	*k*	0.00349	2.0E−4	3.0E−3	3.9E−3
	*n*	1.0039	0.0146	0.9753	1.0324
Bridges	*A*	64.0	0.9422	62.2	65.9
	$W_{0}$	3.06	0.0967	2.87	3.25
	*k*	0.00349	2.0E−4	3.0E−3	3.9E−3
	*n*	1.0038	0.0146	0.9753	1.0324
Generalized Weibull	*A*	67.1	0.9267	65.3	68.9
	$W_{0}$	3.06	0.0996	2.86	3.26
	*k*	0.00356	1.0E−4	3.4E−3	3.8E−3
	*n*	1.0038	0.0157	0.9730	1.0346

a *A* = asymptotic (mature) weight; *W*_*0*_ = initial (birth) weight; *k* = maturation rate parameter; *K* = age at which 50% of asymptotic weight is achieved (for Michaelis-Menten and Generalized Michaelis-Menten functions).

**Table 4:** Age and weight at inflection derived from parameters estimated for ten non-linear functions fitted to describe the growth curve of Boer goats from the Mexican national breeding flock.

Model	Parameter^a^	Expression for calculation^b^	Parameter value
Brody	$t_{i}$	–	–
	$W_{i}$	–	–
Logistic	$t_{i}$	$\left(\frac{1}{k}\right)\ln\left(\frac{A}{W_{0}}-1\right)$	115.1 ± 0.9
	$W_{i}$	$\frac{A}{2}$	24.8 ± 0.3
von Bertalanffy	$t_{i}$	$\left(\frac{1}{k}\right)\ln\left\{3\left[1-{\left(\frac{W_{0}}{A}\right)}^{\frac{1}{3}}\right]\right\}$	74.9 ± 0.7
	$W_{i}$	$\left(\frac{8}{27}\right)A$	17.0 ± 0.2
Gompertz	$t_{i}$	$\left(\frac{1}{k}\right)\ln\left[\ln\left(\frac{A}{W_{0}}\right)\right]$	92.0 ± 0.7
	$W_{i}$	$\frac{A}{e}$	20.0 ± 0.2
Michaelis-Menten	$t_{i}$	–	–
	$W_{i}$	–	–
Generalized Michaelis-Menten	$t_{i}$	$K{\left(\frac{n-1}{n+1}\right)}^{\frac{1}{n}}$	293.0 ± 4.5
	$W_{i}$	$\frac{\left[\left(1+\frac{1}{n}\right)W_{0}+\left(1-\frac{1}{n}\right)A\right]}{2}$	11.0 ± 0.4
Richards	$t_{i}$	$\left(\frac{1}{k}\right)\ln\left\{n\left[1-{\left(\frac{W_{0}}{A}\right)}^{\frac{1}{n}}\right]\right\}$	51.5 ± 2.1
	$W_{i}$	$A{\left(\frac{n-1}{n}\right)}^{n}$	12.9 ± 0.4
Janoscheck	$t_{i}$	–	–
	$W_{i}$	–	–
Bridges	$t_{i}$	–	–
	$W_{i}$	–	–
Generalized Weibull	$t_{i}$	–	–
	$W_{i}$	–	–

a $t_{i}$ = age at inflection (days); $W_{i}$ = weight at inflection (kg).

b *A* = asymptotic (mature) weight; *W*_*0*_ = initial (birth) weight; *k* = maturation rate parameter; *n* = inflection parameter; *ln* = natural logarithm; *K* = age at which 50% of asymptotic weight is achieved (for Michaelis-Menten and Generalized Michaelis-Menten functions).

**Table 5:** Estimates of variance for asymptotic (mature) weight and residual variance, after fitting ten non-linear functions to describe the growth curve of Boer goats from the Mexican national breeding flock.

Model	Parameter	Estimate	Std. Error	95% Confidence Limits	
Brody	$\sigma_{A}^{2}$	160.3	8.8075	143.0	177.5
	$\sigma_{e}^{2}$	9.08	0.2457	8.60	9.57
Logistic	$\sigma_{A}^{2}$	246.3	13.3308	220.1	272.4
	$\sigma_{e}^{2}$	9.07	0.2428	8.59	9.54
Von Bertalanffy	$\sigma_{A}^{2}$	164.1	8.5240	147.4	180.9
	$\sigma_{e}^{2}$	8.63	0.2327	8.17	9.08
Gompertz	$\sigma_{A}^{2}$	182.3	9.4439	163.8	200.8
	$\sigma_{e}^{2}$	8.41	0.2269	8.00	8.90
Michaelis-Menten	$\sigma_{A}^{2}$	294.3	16.9740	261.0	327.7
	$\sigma_{e}^{2}$	8.68	0.2351	8.22	9.14
Generalized Michaelis-Menten	$\sigma_{A}^{2}$	189.9	11.0145	168.3	211.6
	$\sigma_{e}^{2}$	8.21	0.2221	7.78	8.65
Richards	$\sigma_{A}^{2}$	179.8	10.0324	160.0	199.0
	$\sigma_{e}^{2}$	8.64	0.2333	8.20	9.10
Janoscheck	$\sigma_{A}^{2}$	159.6	9.2122	141.5	177.6
	$\sigma_{e}^{2}$	9.09	0.2456	8.60	9.60
Bridges	$\sigma_{A}^{2}$	159.6	9.2247	141.5	177.7
	$\sigma_{e}^{2}$	9.09	0.2457	8.60	9.60
Generalized Weibull	$\sigma_{A}^{2}$	159.6	9.1388	141.6	177.5
	$\sigma_{e}^{2}$	9.09	0.2456	8.60	9.60

**Table 6:** Model ranking and model goodness of fit estimators after fitting ten non-linear functions to describe the growth curve of Boer goats from the Mexican national breeding flock.

Model	Model ranking with:			-2 Log Likelihood	AIC^a^	BIC^b^	Sy/x^c^
	AIC	BIC	Sy/x				
Brody	5	5	2	21,292	21,302	21,327	5.26
Logistic	6	7	7	21,622	21,632	21,657	6.77
Von Bertalanffy	4	4	5	21,284	21,294	21,319	5.80
Gompertz	5	5	6	21,291	21,301	21,325	6.09
Michaelis-Menten	2	2	3	21,204	21,214	21,239	5.31
Generalized Michaelis-Menten	1	1	1	21,012	21,024	21,054	5.22
Richards	3	3	4	21,230	21,242	21,272	5.60
Janoschek	5	6	2	21,292	21,304	21,334	5.26
Bridges	5	6	2	21,292	21,304	21,334	5.26
Generalized Weibull	5	6	2	21,292	21,304	21,334	5.26

a AIC = Akaike Information Criterion.

b BIC = Bayesian Information Criterion.

c Sy/x = Standard error of the regression.

### 2.3 High resolution plots

**Fig. 2:**
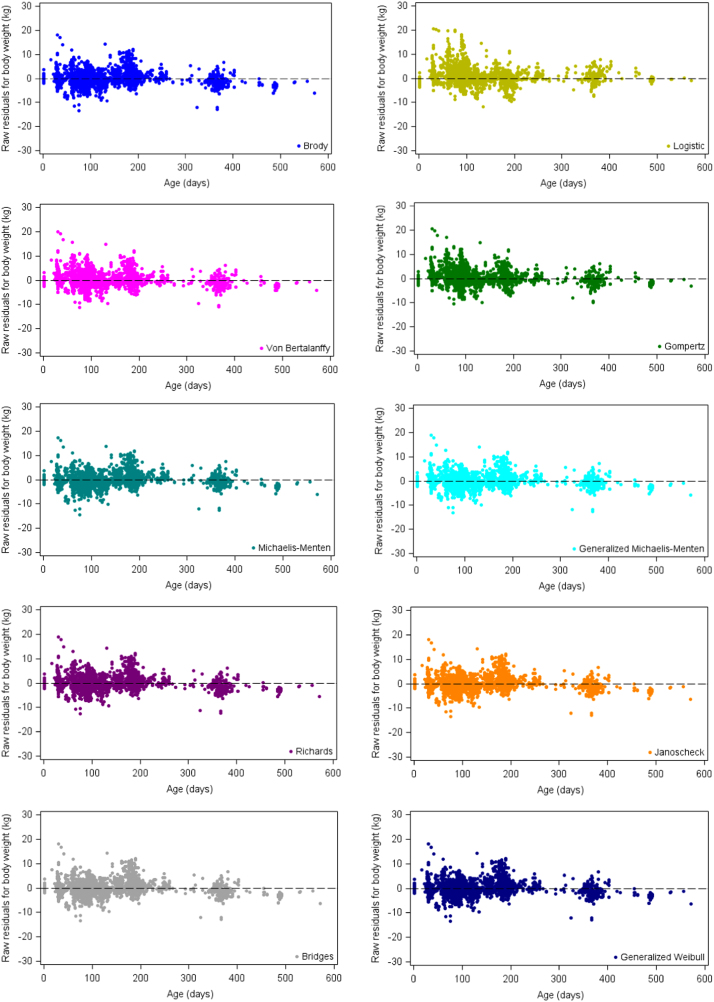
Visual display of raw residuals for body weight (kg) plotted against age (days) of growth data from Boer goats fitted by 10 non-linear functions.

The SAS code (Appendix B) included an expression to calculate the predicted values for the fixed average growth curve as well as the residuals for each of the ten growth functions fitted. The SGPLOT procedure of SAS [8] was used to produce the high-resolution plots displaying the average growth curves (Fig. 1) and the residual plots (Fig. 2). The ***A*** parameter calculated for the fixed regression curve of each function was included as a constant in the SAS code to produce the horizontal asymptote of the respective growth curve plots.
