# Supplementary material for: Dataset on growth curves of Boer goats fitted by ten non-linear functions
Source: Data Brief. 2019 Jan 15;23:103672. doi: 10.1016/j.dib.2019.01.020 (PMC6373209; doi:10.1016/j.dib.2019.01.020)
Supplement: Supplementary file 3 — Supplementary material [file mmc3.docx]

**Appendix B. SAS code used to fit the ten growth functions**

Title 'Brody function';

Options ls=**120** PS=**500** nocenter nodate;

**Proc** **sort** data=Boer; by Animal t;

**run**;**quit**;

**Proc** **nlmixed** data=Boer cov corr technique=DBLDOG method=FIRO maxiter=**200**;

parms A=**50** to **100** by **10** W0=**1** to **5** by **0.5** k=**0.0001** to **0.0006** by **0.0001** s2a1=**550** s2e=**15**/best=**4**;

bounds s2a1 >= **0**, s2e > **0**;

y = (A+a1)-((A+a1)-W0)*exp(-k*t);

xb = A-(A-W0)*exp(-k*t);

model Weight ~ normal(y,s2e);

random a1 ~ normal(**0**,s2a1) subject=Animal;

predict y out=output_randomBRO;

predict xb out=output_fixedBRO;

**run**;**quit**;

/*Residual plot-Brody*/

**data** residualsBRO; set output_randomBRO;

ResidualW=Weight-Pred;

where Weight NE **.**;

**run**;**quit**;

title;

**proc** **sort** data=residualsBRO; by t; **run**; **quit**;

ods graphics on/width=**14** cm height=**8** cm noborder;

**proc** **sgplot** data=residualsBRO noautolegend;

scatter x=t y=ResidualW/MARKERATTRS=(color="black" symbol=circlefilled size=**6** color=blue) legendlabel="Brody" name='BRO';

xaxis VALUEATTRS=(size=**12**) label="Age (days)" values = (**0** to **600** by **100**) LABELATTRS=(size=**12**);

yaxis VALUEATTRS=(size=**12**) label='Raw residuals for body weight (kg)' values = (-**30** to **30** by **10**) LABELATTRS=(size=**12**);

keylegend 'BRO'/ location=inside position=bottomright noborder across=**1** VALUEATTRS=(size=**12**);

refline **0**/axis=y lineattrs=(color="black" pattern=**4** Thickness=**1**);

**run**;**quit**;

/*********Programs to merge files with individual and mean predicted values******/

Goptions reset=all;

Options ls=**256** PS=**5000** nocenter;

**data** output_randomBRO;

set output_randomBRO (keep=Animal t Weight Pred);

rename Pred=PredindBRO; rename Weight=WeightBRO;

**proc** **sort** data=output_randomBRO;

by Animal t;

**run**;**quit**;

**data** output_fixedBRO;

set output_fixedBRO (keep=Animal t Weight Pred);

rename Pred=PredmeanBRO; rename Weight=WeightBRO;

**proc** **sort** data=output_fixedBRO;

by Animal t;

**run**;**quit**;

**data** bothPredictedBRO;

merge output_randomBRO output_fixedBRO;

by Animal t;

**run**;**quit**;

**proc** **sort** data=bothPredictedBRO;

by t;

**run**;**quit**;

ods graphics on /width=**14** cm height=**8** cm noborder;

**proc** **sgplot** data=bothPredictedBRO noautolegend;

series x=t y=PredindBRO/group=Animal lineattrs=(pattern=2 color=red thickness=0.5);

scatter x=t y=WeightBRO/MARKERATTRS=(color="black" symbol=circlefilled size=**4** color=black);

series x=t y=PredmeanBRO/ lineattrs=(pattern=**1** color=blue thickness=**5**) legendlabel="Brody" name='BRO';

xaxis VALUEATTRS=(size=**12**) label="Age (days)" values = (**0** to **2190** by **365**) LABELATTRS=(size=**12**);

yaxis VALUEATTRS=(size=**12**) label='Body weight (kg)' values = (**0** to **100** by **25**) LABELATTRS=(size=**12**);

keylegend 'BRO'/ location=inside position=bottomright noborder across=**1** VALUEATTRS=(size=**12**);

refline **67.28**/axis=y lineattrs=(color="black" pattern=**4** Thickness=**2**);

**run**;**quit**;

Title 'Logistic function';

Options ls=**120** PS=**500** nocenter nodate;

**Proc** **sort** data=Boer; by Animal t;

**run**;**quit**;

**Proc** **nlmixed** data=Boer cov corr technique=DBLDOG method=FIRO maxiter=**200**;

parms A=**50** to **100** by **10** W0=**1** to **5** by **1** k=**1** to 15 by **5** s2a1=**550** s2e=**15**/best=**4**;

bounds s2a1 >= **0**, s2e > **0**;

y =(A+a1)*(**1**+(((A+a1)-W0)/W0)*exp(-k*t))**-**1**;

xb=A*(**1**+((A-W0)/W0)*exp(-k*t))**-**1**;

model Weight ~ normal(y,s2e);

random a1 ~ normal(**0**,s2a1) subject=Animal;

predict y out=output_randomLOG;

predict xb out=output_fixedLOG;

estimate 'TimeInflxn' (**1**/k)*log((A/W0)-**1**);

estimate 'WgInflxn' A/**2**;

**run**;**quit**;

/*Residual plot-Logistic*/

**data** residualsLOG; set output_randomLOG;

ResidualW=Weight-Pred;

where Weight NE **.**;

**run**;**quit**;

title;

**proc** **sort** data=residualsLOG; by t; **run**; **quit**;

ods graphics on /width=**14** cm height=**8** cm noborder;

**proc** **sgplot** data=residualsLOG noautolegend;

scatter x=t y=ResidualW/MARKERATTRS=(color="black" symbol=circlefilled size=**6** color=blue) legendlabel="Logistic" name='LOG';

xaxis VALUEATTRS=(size=**12**) label="Age (days)" values = (**0** to **600** by **100**) LABELATTRS=(size=**12**);

yaxis VALUEATTRS=(size=**12**) label='Raw residuals for body weight (kg)' values = (-**30** to **30** by **10**) LABELATTRS=(size=**12**);

keylegend 'LOG'/ location=inside position=bottomright noborder across=**1** VALUEATTRS=(size=**12**);

refline **0**/axis=y lineattrs=(color="black" pattern=**4** Thickness=**1**);

**run**;**quit**;

/*********Programs to merge files with individual and mean predicted values******/

Goptions reset=all;

Options ls=**256** PS=**5000** nocenter;

**data** output_randomLOG;

set output_randomLOG (keep=Animal t Weight Pred);

rename Pred=PredindLOG; rename Weight=WeightLOG;

**proc** **sort** data=output_randomLOG;

by Animal t;

**run**;**quit**;

**data** output_fixedLOG;

set output_fixedLOG (keep=Animal t Weight Pred);

rename Pred=PredmeanLOG; rename Weight=WeightLOG;

**proc** **sort** data=output_fixedLOG;

by Animal t;

**run**;**quit**;

**data** bothPredictedLOG;

merge output_randomLOG output_fixedLOG;

by Animal t;

**run**;**quit**;

**proc** **sort** data=bothPredictedLOG;

by t;

**run**;**quit**;

ods graphics on /width=**14** cm height=**8** cm noborder;

**proc** **sgplot** data=bothPredictedLOG noautolegend;

series x=t y=PredindLOG/group=Animal lineattrs=(pattern=2 color=red thickness=0.5);

scatter x=t y=WeightLOG/MARKERATTRS=(color="black" symbol=circlefilled size=**4** color=black);

series x=t y=PredmeanLOG/ lineattrs=(pattern=**1** color=blue thickness=**5**) legendlabel="Logistic" name='LOG';

xaxis VALUEATTRS=(size=**12**) label="Age (days)" values = (**0** to **2190** by **365**) LABELATTRS=(size=**12**);

yaxis VALUEATTRS=(size=**12**) label='Body weight (kg)' values = (**0** to **100** by **25**) LABELATTRS=(size=**12**);

keylegend 'LOG'/ location=inside position=bottomright noborder across=**1** VALUEATTRS=(size=**12**);

refline **49.5234**/axis=y lineattrs=(color="black" pattern=**4** Thickness=**2**);

**run**;**quit**;

Title 'Von Bertalanffy function';

Options ls=**120** PS=**500** nocenter nodate;

**Proc** **sort** data=Boer; by Animal t;

**run**;**quit**;

**Proc** **nlmixed** data=Boer cov corr technique=DBLDOG method=FIRO maxiter=**200**;

parms A=**50** to **100** by **10** W0=**1** to **5** by **1** k=**0.001** to **0.003** by **0.001** s2a1=**550** s2e=**15**/best=**4**;

bounds s2a1 >= **0**, s2e > **0**;

y = (A+a1)*(**1**-(**1**-((W0/(A+a1))**(**1**/**3**)))*exp(-k*t))****3**;

xb = A*(**1**-(**1**-((W0/A)**(**1**/**3**)))*exp(-k*t))****3**;

model Weight ~ normal(y,s2e);

random a1 ~ normal(**0**,s2a1) subject=Animal;

predict y out=output_randomVB;

predict xb out=output_fixedVB;

estimate 'TimeInflxn' (**1**/k)*log(**3***(**1**-(W0/A)**(**1**/**3**)));

estimate 'WgInflxn' (**8**/**27**)*A;

**run**;**quit**;

/*Residual plot-Von Bertalanffy*/

**data** residualsVB; set output_randomVB;

ResidualW=Weight-Pred;

where Weight NE **.**;

**run**;**quit**;

title;

**proc** **sort** data=residualsVB; by t; **run**; **quit**;

ods graphics on/width=**14** cm height=**8** cm noborder;

**proc** **sgplot** data=residualsVB noautolegend;

scatter x=t y=ResidualW/MARKERATTRS=(color="black" symbol=circlefilled size=**6** color=blue) legendlabel="Von Bertalanffy" name='VB';

xaxis VALUEATTRS=(size=**12**) label="Age (days)" values = (**0** to **600** by **100**) LABELATTRS=(size=**12**);

yaxis VALUEATTRS=(size=**12**) label='Raw residuals for body weight (kg)' values = (-**30** to **30** by **10**) LABELATTRS=(size=**12**);

keylegend 'VB'/ location=inside position=bottomright noborder across=**1** VALUEATTRS=(size=**12**);

refline **0**/axis=y lineattrs=(color="black" pattern=**4** Thickness=**1**);

**run**;**quit**;

/*********Programs to merge files with individual and mean predicted values******/

Goptions reset=all;

Options ls=**256** PS=**5000** nocenter;

**data** output_randomVB;

set output_randomVB (keep=Animal t Weight Pred);

rename Pred=PredindVB; rename Weight=WeightVB;

**proc** **sort** data=output_randomVB;

by Animal t;

**run**;**quit**;

**data** output_fixedVB;

set output_fixedVB (keep=Animal t Weight Pred);

rename Pred=PredmeanVB; rename Weight=WeightVB;

**proc** **sort** data=output_fixedVB;

by Animal t;

**run**;**quit**;

**data** bothPredictedVB;

merge output_randomVB output_fixedVB;

by Animal t;

**run**;**quit**;

**proc** **sort** data=bothPredictedVB;

by t;

**run**;**quit**;

ods graphics on /width=**14** cm height=**8** cm noborder;

**proc** **sgplot** data=bothPredictedVB noautolegend;

series x=t y=PredindVB/group=Animal lineattrs=(pattern=2 color=red thickness=0.5);

scatter x=t y=WeightVB/MARKERATTRS=(color="black" symbol=circlefilled size=**4** color=black);

series x=t y=PredmeanVB/ lineattrs=(pattern=**1** color=blue thickness=**5**) legendlabel="Von Bertalanffy" name='VB';

xaxis VALUEATTRS=(size=**12**) label="Age (days)" values = (**0** to **2190** by **365**) LABELATTRS=(size=**12**);

yaxis VALUEATTRS=(size=**12**) label='Body weight (kg)' values = (**0** to **100** by **25**) LABELATTRS=(size=**12**);

keylegend 'VB'/ location=inside position=bottomright noborder across=**1** VALUEATTRS=(size=**12**);

refline **57.3766**/axis=y lineattrs=(color="black" pattern=**4** Thickness=**2**);

**run**;**quit**;

Title 'Gompertz function';

Options ls=**120** PS=**500** nocenter nodate;

**Proc** **sort** data=Boer; by Animal t;

**run**;**quit**;

**Proc** **nlmixed** data=Boer cov corr technique=DBLDOG method=FIRO maxiter=**200**;

parms A=**50** to **100** by **10** W0=**1** to **5** by **1** k=**0.001** to **0.003** by **0.001** s2a1=**550** s2e=**15**/best=**4**;

bounds s2a1 >= **0**, s2e > **0**;

y = (A+a1)*(W0/(A+a1))**exp(-k*t);

xb = A*(W0/A)**exp(-k*t);

model Weight ~ normal(y,s2e);

random a1 ~ normal(**0**,s2a1) subject=Animal;

predict y out=output_randomGOM;

predict xb out=output_fixedGOM;

estimate 'TimeInflxn' (**1**/k)*log(log(A/W0));

estimate 'WgInflxn' (A/**2.71828182**);

**run**;**quit**;

/*Residual plot-Gompertz*/

**data** residualsGOM; set output_randomGOM;

ResidualW=Weight-Pred;

where Weight NE **.**;

**run**;**quit**;

title;

**proc** **sort** data=residualsGOM; by t; **run**; **quit**;

ods graphics on /width=**14** cm height=**8** cm noborder;

**proc** **sgplot** data=residualsGOM noautolegend;

scatter x=t y=ResidualW/MARKERATTRS=(color="black" symbol=circlefilled size=**6** color=blue) legendlabel="Gompertz" name='GOM';

xaxis VALUEATTRS=(size=**12**) label="Age (days)" values = (**0** to **600** by **100**) LABELATTRS=(size=**12**);

yaxis VALUEATTRS=(size=**12**) label='Raw residuals for body weight (kg)' values = (-**30** to **30** by **10**) LABELATTRS=(size=**12**);

keylegend 'GOM'/ location=inside position=bottomright noborder across=**1** VALUEATTRS=(size=**12**);

refline **0**/axis=y lineattrs=(color="black" pattern=**4** Thickness=**1**);

**run**;**quit**;

/*********Programs to merge files with individual and mean predicted values******/

Goptions reset=all;

Options ls=**256** PS=**5000** nocenter;

**data** output_randomGOM;

set output_randomGOM (keep=Animal t Weight Pred);

rename Pred=PredindGOM; rename Weight=WeightGOM;

**proc** **sort** data=output_randomGOM;

by Animal t;

**run**;**quit**;

**data** output_fixedGOM;

set output_fixedGOM (keep=Animal t Weight Pred);

rename Pred=PredmeanGOM; rename Weight=WeightGOM;

**proc** **sort** data=output_fixedGOM;

by Animal t;

**run**;**quit**;

**data** bothPredictedGOM;

merge output_randomGOM output_fixedGOM;

by Animal t;

**run**;**quit**;

**proc** **sort** data=bothPredictedGOM;

by t;

**run**;**quit**;

ods graphics on /width=**14** cm height=**8** cm noborder;

**proc** **sgplot** data=bothPredictedGOM noautolegend;

series x=t y=PredindGOM/group=Animal lineattrs=(pattern=2 color=red thickness=0.5);

scatter x=t y=WeightGOM/MARKERATTRS=(color="black" symbol=circlefilled size=**4** color=black);

series x=t y=PredmeanGOM/ lineattrs=(pattern=**1** color=blue thickness=**5**) legendlabel="Gompertz" name='GOM';

xaxis VALUEATTRS=(size=**12**) label="Age (days)" values = (**0** to **2190** by **365**) LABELATTRS=(size=**12**);

yaxis VALUEATTRS=(size=**12**) label='Body weight (kg)' values = (**0** to **100** by **25**) LABELATTRS=(size=**12**);

keylegend 'GOM'/ location=inside position=bottomright noborder across=**1** VALUEATTRS=(size=**12**);

refline **54.7324**/axis=y lineattrs=(color="black" pattern=**4** Thickness=**2**);

**run**;**quit**;

Title 'Michaelis-Menten function';

Options ls=**120** PS=**500** nocenter nodate;

**Proc** **sort** data=Boer; by Animal t;

**run**;**quit**;

**Proc** **nlmixed** data=Boer cov corr technique=DBLDOG method=FIRO maxiter=**200**;

parms A=**50** to **100** by **10** W0=**1** to **5** by **1** K=**100** to **300** by **100** s2a1=**550** s2e=**15**/best=**4**;

bounds s2a1 >= **0**, s2e > **0, K > 0** ;

y = ((W0*K****1**+((A+a1)*t****1**)))/((K****1**)+(t****1**));

xb = ((W0*K****1**)+(A*t****1**))/((K****1**)+(t****1**));

model Weight ~ normal(y,s2e);

random a1 ~ normal(**0**,s2a1) subject=Animal;

predict y out=output_randomMM;

predict xb out=output_fixedMM;

**run**;**quit**;

/*Residual plot-Michaelis-Menten*/

**data** residualsMM; set output_randomMM;

ResidualW=Weight-Pred;

where Weight NE **.**;

**run**;**quit**;

title;

**proc** **sort** data=residualsMM; by t; **run**; **quit**;

ods graphics on/width=**14** cm height=**8** cm noborder;

**proc** **sgplot** data=residualsMM noautolegend;

scatter x=t y=ResidualW/MARKERATTRS=(color="black" symbol=circlefilled size=**6** color=blue) legendlabel="Michaelis-Menten" name='MM';

xaxis VALUEATTRS=(size=**12**) label="Age (days)" values = (**0** to **600** by **100**) LABELATTRS=(size=**12**);

yaxis VALUEATTRS=(size=**12**) label='Raw residuals for body weight (kg)' values = (-**30** to **30** by **10**) LABELATTRS=(size=**12**);

keylegend 'MM'/ location=inside position=bottomright noborder across=**1** VALUEATTRS=(size=**12**);

refline **0**/axis=y lineattrs=(color="black" pattern=**4** Thickness=**1**);

**run**;**quit**;

/*********Programs to merge files with individual and mean predicted values******/

Goptions reset=all;

Options ls=**256** PS=**5000** nocenter;

**data** output_randomMM;

set output_randomMM (keep=Animal t Weight Pred);

rename Pred=PredindMM; rename Weight=WeightMM;

**proc** **sort** data=output_randomMM;

by Animal t;

**run**;**quit**;

**data** output_fixedMM;

set output_fixedMM (keep=Animal t Weight Pred);

rename Pred=PredmeanMM; rename Weight=WeightMM;

**proc** **sort** data=output_fixedMM;

by Animal t;

**run**;**quit**;

**data** bothPredictedMM;

merge output_randomMM output_fixedMM;

by Animal t;

**run**;**quit**;

**proc** **sort** data=bothPredictedMM;

by t;

**run**;**quit**;

ods graphics on /width=**14** cm height=**8** cm noborder;

**proc** **sgplot** data=bothPredictedMM noautolegend;

series x=t y=PredindMM/group=Animal lineattrs=(pattern=2 color=red thickness=0.5);

scatter x=t y=WeightMM/MARKERATTRS=(color="black" symbol=circlefilled size=**4** color=black);

series x=t y=PredmeanMM/ lineattrs=(pattern=**1** color=blue thickness=**5**) legendlabel=" Michaelis-Menten " name='MM';

xaxis VALUEATTRS=(size=**12**) label="Age (days)" values = (**0** to **2190** by **365**) LABELATTRS=(size=**12**);

yaxis VALUEATTRS=(size=**12**) label='Body weight (kg)' values = (**0** to **100** by **25**) LABELATTRS=(size=**12**);

keylegend 'MM'/ location=inside position=bottomright noborder across=**1** VALUEATTRS=(size=**12**);

refline **88.4660**/axis=y lineattrs=(color="black" pattern=**4** Thickness=**2**);

**run**;**quit**;

Title 'Generalized Michaelis-Menten function';

**Proc** **sort** data=Boer;

by Animal t;

**run**;**quit**;

**Proc** **nlmixed** data=Boer cov corr technique=DBLDOG METHOD=firo maxiter=**100**;

parms A=**50** to **100** by **10** W0=**1** to **5** by **1**  K=**1** to **3** by **1** n=**1** to **5** by **1** s2a1=**550** s2e=**150**/best=**4**;

bounds s2a1 >= **0**, s2e > **0**, n > **0**, K > **0**;

y = ((W0*K**n+((A+a1)*t**n)))/((K**n)+(t**n));

xb = ((W0*K**n)+(A*t**n))/((K**n)+(t**n));

model Peso ~ normal(y,s2e);

random a1 ~ normal(**0**,s2a1) subject=Animal;

predict xb out=output_fixedGMM;

predict y out=output_randomGMM;

estimate 'TimeInflxn' K*((n-**1**/n+**1**)**(**1**/n));

estimate 'WgInflxn' (W0*((**1**)+(**1**/n))+A*((**1**)-(**1**/n)))/**2**;

**run**;**quit**;

/*Residual plot-Generalized Michaelis-Menten*/

**data** residualesGMM; set output_randomGMM;

ResidualW=Peso-Pred;

where Peso NE **.**;

**run**;**quit**;

title;

**proc** **sort** data=residualesGMM; by t; **run**; **quit**;

ods graphics on/width=**14** cm height=**8** cm noborder;

**proc** **sgplot** data=residualesGMM noautolegend;

scatter x=t y=ResidualW/MARKERATTRS=(color="black" symbol=circlefilled size=**6** color="cyan") legendlabel="Generalized Michaelis-Menten" name='GMM';

xaxis VALUEATTRS=(size=**12**) label="Age (days)" values = (**0** to **600** by **100**) LABELATTRS=(size=**12**);

yaxis VALUEATTRS=(size=**12**) label='Raw residuals for body weight (kg)' values = (-**30** to **30** by **10**) LABELATTRS=(size=**12**);

keylegend 'GMM'/ location=inside position=bottomright noborder across=**1** VALUEATTRS=(size=**12**);

refline **0**/axis=y lineattrs=(color="black" pattern=**4** Thickness=**1**);

**run**;**quit**;

/*********Programs to merge files with individual and mean predicted values******/

Goptions reset=all;

Options ls=**256** PS=**5000** nocenter;

**data** output_randomGMM;

set output_randomGMM (keep=Animal t Weight Pred);

rename Pred=PredindGMM; rename Weight = WeightGMM;

**proc** **sort** data=output_randomGMM;

by Animal t;

**run**;**quit**;

**data** output_fixedGMM;

set output_fixedGMM (keep=Animal t Weight Pred);

rename Pred=PredmeanGMM; rename Weight=WeightGMM;

**proc** **sort** data=output_fixedGMM;

by Animal t;

**run**;**quit**;

**data** bothPredictedGMM;

merge output_randomGMM output_fixedGMM;

by Animal t;

**run**;**quit**;

**proc** **sort** data=bothPredictedGMM;

by t;

**run**;**quit**;

ods graphics on /width=**14** cm height=**8** cm noborder;

**proc** **sgplot** data=bothPredictedGMM noautolegend;

series x=t y=PredindGMM/group=Animal lineattrs=(pattern=2 color=red thickness=0.5);

scatter x=t y=WeightGMM/MARKERATTRS=(color="black" symbol=circlefilled size=**4**);

series x=t y=PredmeanGMM/lineattrs=(pattern=**1** color=cyan thickness=**5**) legendlabel="Generalized Michaelis-Menten"

name='GMM';

xaxis VALUEATTRS=(size=**12**) label="Age (days)" values=(**0** to **2190** by **365**) LABELATTRS=(size=**12**);

yaxis VALUEATTRS=(size=**12**) label='Body weight (kg)' values=(**0** to **100** by **25**) LABELATTRS=(size=**12**);

keylegend 'GMM'/ location=inside position=bottomright noborder across=**1** VALUEATTRS=(size=**12**);

refline **71.8184**/axis=y lineattrs=(color="black" pattern=**4** Thickness=**2**);

**run**;**quit**;

Title 'Richards function';

Options ls=**120** PS=**500** nocenter nodate;

**Proc** **sort** data=Boer; by Animal t;

**run**;**quit**;

**Proc** **nlmixed** data=Boer cov corr technique=DBLDOG method=FIRO maxiter=**200**;

parms A=**50** to **100** by **10** W0=**1** to **5** by **1** k=**0.0001** to **0.0005** by **0.0001** n=1 to 5 by 1 s2a1=**550** s2e=**150**/best=**4**;

bounds s2a1 >= **0**, s2e > **0**;

y = (A+a1)*(**1**-(**1**-(W0/(A+a1)))**(**1**/n)*exp(-k*t))**n;

xb = A*(**1**-(**1**-(W0/A))**(**1**/n)*exp(-k*t))**n;

model Weight ~ normal(y,s2e);

random a1 ~ normal(**0**,s2a1) subject=Animal;

predict y out=output_randomRICH;

predict xb out=output_fixedRICH;

estimate 'TimeInflxn' (**1**/k)*(log(n*(**1**-W0/A)**(**1**/n)));

estimate 'WgInflxn' A*(((n-**1**)/n)**n);

**run**;**quit**;

/*Residual plot-Richards*/

**data** residualsRICH; set output_randomRICH;

ResidualW=Weight-Pred;

where Weight NE **.**;

**run**;**quit**;

title;

**proc** **sort** data=residualsRICH; by t; **run**; **quit**;

ods graphics on /width=**14** cm height=**8** cm noborder;

**proc** **sgplot** data=residualsRICH noautolegend;

scatter x=t y=ResidualW/MARKERATTRS=(color="black" symbol=circlefilled size=**6** color=blue) legendlabel="Richards" name='RICH';

xaxis VALUEATTRS=(size=**12**) label="Age (days)" values = (**0** to **600** by **100**) LABELATTRS=(size=**12**);

yaxis VALUEATTRS=(size=**12**) label='Raw residuals for body weight (kg)' values = (-**30** to **30** by **10**) LABELATTRS=(size=**12**);

keylegend 'RICH'/ location=inside position=bottomright noborder across=**1** VALUEATTRS=(size=**12**);

refline **0**/axis=y lineattrs=(color="black" pattern=**4** Thickness=**1**);

**run**;**quit**;

/*********Programs to merge files with individual and mean predicted values******/

Goptions reset=all;

Options ls=**256** PS=**5000** nocenter;

**data** output_randomRICH;

set output_randomRICH (keep=Animal t Weight Pred);

rename Pred=PredindRICH; rename Weight=WeightRICH;

**proc** **sort** data=output_randomRICH;

by Animal t;

**run**;**quit**;

**data** output_fixedRICH;

set output_fixedRICH (keep=Animal t Weight Pred);

rename Pred=PredmeanRICH; rename Weight=WeightRICH;

**proc** **sort** data=output_fixedRICH;

by Animal t;

**run**;**quit**;

**data** bothPredictedRICH;

merge output_randomRICH output_fixedRICH;

by Animal t;

**run**;**quit**;

**proc** **sort** data=bothPredictedRICH;

by t;

**run**;**quit**;

ods graphics on /width=**14** cm height=**8** cm noborder;

**proc** **sgplot** data=bothPredictedRICH noautolegend;

series x=t y=PredindRICH/group=Animal lineattrs=(pattern=2 color=red thickness=0.5);

scatter x=t y=WeightRICH/MARKERATTRS=(color="black" symbol=circlefilled size=**4** color=black);

series x=t y=PredmeanRICH/ lineattrs=(pattern=**1** color=blue thickness=**5**) legendlabel="Richards" name='RICH';

xaxis VALUEATTRS=(size=**12**) label="Age (days)" values = (**0** to **2190** by **365**) LABELATTRS=(size=**12**);

yaxis VALUEATTRS=(size=**12**) label='Body weight (kg)' values = (**0** to **100** by **25**) LABELATTRS=(size=**12**);

keylegend 'RICH'/ location=inside position=bottomright noborder across=**1** VALUEATTRS=(size=**12**);

refline **59.4904**/axis=y lineattrs=(color="black" pattern=**4** Thickness=**2**);

**run**;**quit**;

Title 'Janoscheck function';

Options ls=**120** PS=**500** nocenter nodate;

**Proc** **sort** data=Boer; by Animal t;

**run**;**quit**;

**Proc** **nlmixed** data=Boer cov corr technique=DBLDOG method=FIRO maxiter=**200**;

parms A=**50** to **100** by **10** W0=**1** to **5** by **1** k=**0.0001** to **0.0003** by **0.0001** n=1 to 5 by 1 s2a1=**550** s2e=**15**/best=**4**;

bounds s2a1 >= **0**, s2e > **0**;

y = (A+a1)-((A+a1)-W0)*(exp(-k*t**n));

xb = A-(A-W0)*(exp(-k*t**n));

model Weight ~ normal(y,s2e);

random a1 ~ normal(**0**,s2a1) subject=Animal;

predict y out=output_randomJAN;

predict xb out=output_fixedJAN;

**run**;**quit**;

/*Residual plot-Janoscheck*/

**data** residualsJAN; set output_randomJAN;

ResidualW=Weight-Pred;

where Weight NE **.**;

**run**;**quit**;

title;

**proc** **sort** data=residualsJAN; by t; **run**; **quit**;

ods graphics on /width=**14** cm height=**8** cm noborder;

**proc** **sgplot** data=residualsJAN noautolegend;

scatter x=t y=ResidualW/MARKERATTRS=(color="black" symbol=circlefilled size=**6** color=blue) legendlabel="Janoscheck" name='JAN';

xaxis VALUEATTRS=(size=**12**) label="Age (days)" values = (**0** to **600** by **100**) LABELATTRS=(size=**12**);

yaxis VALUEATTRS=(size=**12**) label='Raw residuals for body weight (kg)' values = (-**30** to **30** by **10**) LABELATTRS=(size=**12**);

keylegend 'JAN'/ location=inside position=bottomright noborder across=**1** VALUEATTRS=(size=**12**);

refline **0**/axis=y lineattrs=(color="black" pattern=**4** Thickness=**1**);

**run**;**quit**;

/*********Programs to merge files with individual and mean predicted values******/

Goptions reset=all;

Options ls=**256** PS=**5000** nocenter;

**data** output_randomJAN;

set output_randomJAN (keep=Animal t Weight Pred);

rename Pred=PredindJAN; rename Weight=WeightJAN;

**proc** **sort** data=output_randomJAN;

by Animal t;

**run**;**quit**;

**data** output_fixedJAN;

set output_fixedJAN (keep=Animal t Weight Pred);

rename Pred=PredmeanJAN; rename Weight=WeightJAN;

**proc** **sort** data=output_fixedJAN;

by Animal t;

**run**;**quit**;

**data** bothPredictedJAN;

merge output_randomJAN output_fixedJAN;

by Animal t;

**run**;**quit**;

**proc** **sort** data=bothPredictedJAN;

by t;

**run**;**quit**;

ods graphics on /width=**14** cm height=**8** cm noborder;

**proc** **sgplot** data=bothPredictedJAN noautolegend;

series x=t y=PredindJAN/group=Animal lineattrs=(pattern=2 color=red thickness=0.5);

scatter x=t y=WeightJAN/MARKERATTRS=(color="black" symbol=circlefilled size=**4** color=black);

series x=t y=PredmeanJAN/ lineattrs=(pattern=**1** color=blue thickness=**5**) legendlabel="Janoscheck" name='JAN';

xaxis VALUEATTRS=(size=**12**) label="Age (days)" values = (**0** to **2190** by **365**) LABELATTRS=(size=**12**);

yaxis VALUEATTRS=(size=**12**) label='Body weight (kg)' values = (**0** to **100** by **25**) LABELATTRS=(size=**12**);

keylegend 'JAN'/ location=inside position=bottomright noborder across=**1** VALUEATTRS=(size=**12**);

refline **67.1140**/axis=y lineattrs=(color="black" pattern=**4** Thickness=**2**);

**run**;**quit**;

Title 'Bridges function';

Options ls=**120** PS=**500** nocenter nodate;

**Proc** **sort** data=Boer; by Animal t;

**run**;**quit**;

**Proc** **nlmixed** data=Boer cov corr technique=DBLDOG method=FIRO maxiter=**200**;

parms A=**50** to **100** by **10** W0=**1** to **5** by **1** k=**0.0001** to **0.0003** by **0.0001** n=**1** to **5** by **1** s2a1=**550** s2e=**15**/best=**4**;

bounds s2a1 >= **0**, s2e > **0**;

y = W0+(A+a1)*(**1**-exp(-k*t**n));

xb = W0+A*(**1**-exp(-k*t**n));

model Weight ~ normal(y,s2e);

random a1 ~ normal(**0**,s2a1) subject=Animal;

predict y out=output_randomBRI;

predict xb out=output_fixedBRI;

**run**;**quit**;

/*Residual plot-Bridges*/

**data** residualsBRI; set output_randomBRI;

ResidualW=Weight-Pred;

where Weight NE **.**;

**run**;**quit**;

title;

**proc** **sort** data=residualsBRI; by t; **run**; **quit**;

ods graphics on/width=**14** cm height=**8** cm noborder;

**proc** **sgplot** data=residualsBRI noautolegend;

scatter x=t y=ResidualW/MARKERATTRS=(color="black" symbol=circlefilled size=**6** color=blue) legendlabel="Bridges" name='BRI';

xaxis VALUEATTRS=(size=**12**) label="Age (days)" values = (**0** to **600** by **100**) LABELATTRS=(size=**12**);

yaxis VALUEATTRS=(size=**12**) label='Raw residuals for body weight (kg)' values = (-**30** to **30** by **10**) LABELATTRS=(size=**12**);

keylegend 'BRI'/ location=inside position=bottomright noborder across=**1** VALUEATTRS=(size=**12**);

refline **0**/axis=y lineattrs=(color="black" pattern=**4** Thickness=**1**);

**run**;**quit**;

/*********Programs to merge files with individual and mean predicted values******/

Goptions reset=all;

Options ls=**256** PS=**5000** nocenter;

**data** output_randomBRI;

set output_randomBRI (keep=Animal t Weight Pred);

rename Pred=PredindBRI; rename Weight=WeightBRI;

**proc** **sort** data=output_randomBRI;

by Animal t;

**run**;**quit**;

**data** output_fixedBRI;

set output_fixedBRI (keep=Animal t Weight Pred);

rename Pred=PredmeanBRI; rename Weight=WeightBRI;

**proc** **sort** data=output_fixedBRI;

by Animal t;

**run**;**quit**;

**data** bothPredictedBRI;

merge output_randomBRI output_fixedBRI;

by Animal t;

**run**;**quit**;

**proc** **sort** data=bothPredictedBRI;

by t;

**run**;**quit**;

ods graphics on /width=**14** cm height=**8** cm noborder;

**proc** **sgplot** data=bothPredictedBRI noautolegend;

series x=t y=PredindBRI/group=Animal lineattrs=(pattern=2 color=red thickness=0.5);

scatter x=t y=WeightBRI/MARKERATTRS=(color="black" symbol=circlefilled size=**4** color=black);

series x=t y=PredmeanBRI/ lineattrs=(pattern=**1** color=blue thickness=**5**) legendlabel="Bridges" name='BRI';

xaxis VALUEATTRS=(size=**12**) label="Age (days)" values = (**0** to **2190** by **365**) LABELATTRS=(size=**12**);

yaxis VALUEATTRS=(size=**12**) label='Body weight (kg)' values = (**0** to **100** by **25**) LABELATTRS=(size=**12**);

keylegend 'BRI'/ location=inside position=bottomright noborder across=**1** VALUEATTRS=(size=**12**);

refline **64.0550**/axis=y lineattrs=(color="black" pattern=**4** Thickness=**2**);

**run**;**quit**;

Title 'Generalized Weibull function';

Options ls=**120** PS=**500** nocenter nodate;

**Proc** **sort** data=Boer; by Animal t;

**run**;**quit**;

**Proc** **nlmixed** data=Boer cov corr technique=DBLDOG method=FIRO maxiter=**200**;

parms A=**50** to **100** by **10** W0=**1** to **5** by **1** k=**0.001** to **0.003** by **0.001** n=**1** to **5** by **1** s2a1=**550** s2e=**15**/best=**4**;

bounds s2a1 >= **0**, s2e > **0**;

y = (A+a1)-((A+a1)-W0)*exp(-k*t**n);

xb = A-(A-W0)*exp(-k*t**n);

model Weight ~ normal(y,s2e);

random a1 ~ normal(**0**,s2a1) subject=Animal;

predict y out=output_randomWEI;

predict xb out=output_fixedWEI;

**run**;**quit**;

/*Residual plot-Generalized Weibull*/

**data** residualsWEI; set output_randomWEI;

ResidualW=Weight-Pred;

where Weight NE **.**;

**run**;**quit**;

title;

**proc** **sort** data=residualsWEI; by t; **run**; **quit**;

ods graphics on /width=**14** cm height=**8** cm noborder;

**proc** **sgplot** data=residualsWEI noautolegend;

scatter x=t y=ResidualW/MARKERATTRS=(color="black" symbol=circlefilled size=**6** color=blue) legendlabel="Generalized Weibull” name='WEI';

xaxis VALUEATTRS=(size=**12**) label="Age (days)" values = (**0** to **600** by **100**) LABELATTRS=(size=**12**);

yaxis VALUEATTRS=(size=**12**) label='Raw residuals for body weight (kg)' values = (-**30** to **30** by **10**) LABELATTRS=(size=**12**);

keylegend 'WEI'/ location=inside position=bottomright noborder across=**1** VALUEATTRS=(size=**12**);

refline **0**/axis=y lineattrs=(color="black" pattern=**4** Thickness=**1**);

**run**;**quit**;

/*********Programs to merge files with individual and mean predicted values******/

Goptions reset=all;

Options ls=**256** PS=**5000** nocenter;

**data** output_randomWEI;

set output_randomWEI (keep=Animal t Weight Pred);

rename Pred=PredindWEI; rename Weight=WeightWEI;

**proc** **sort** data=output_randomWEI;

by Animal t;

**run**;**quit**;

**data** output_fixedWEI;

set output_fixedWEI (keep=Animal t Weight Pred);

rename Pred=PredmeanWEI; rename Weight=WeightWEI;

**proc** **sort** data=output_fixedWEI;

by Animal t;

**run**;**quit**;

**data** bothPredictedWEI;

merge output_randomWEI output_fixedWEI;

by Animal t;

**run**;**quit**;

**proc** **sort** data=bothPredictedWEI;

by t;

**run**;**quit**;

ods graphics on /width=**14** cm height=**8** cm noborder;

**proc** **sgplot** data=bothPredictedWEI noautolegend;

series x=t y=PredindWEI/group=Animal lineattrs=(pattern=2 color=red thickness=0.5);

scatter x=t y=WeightWEI/MARKERATTRS=(color="black" symbol=circlefilled size=**4** color=black);

series x=t y=PredmeanWEI/ lineattrs=(pattern=**1** color=blue thickness=**5**) legendlabel="Generalized Weibull" name='WEI';

xaxis VALUEATTRS=(size=**12**) label="Age (days)" values = (**0** to **2190** by **365**) LABELATTRS=(size=**12**);

yaxis VALUEATTRS=(size=**12**) label='Body weight (kg)' values = (**0** to **100** by **25**) LABELATTRS=(size=**12**);

keylegend 'WEI'/ location=inside position=bottomright noborder across=**1** VALUEATTRS=(size=**12**);

refline **67.1171**/axis=y lineattrs=(color="black" pattern=**4** Thickness=**2**);

**run**;**quit**;
